# Supplementary material for: Dynamical signatures of molecular symmetries in nonequilibrium quantum transport
Source: Sci Rep. 2016 Jun 17;6:28027. doi: 10.1038/srep28027 (PMC4911572; doi:10.1038/srep28027)
Supplement: Supplementary Information [file srep28027-s1.pdf]

Supplemental material for
'Dynamical signatures of molecular symmetries in nonequilibrium quantum transport'

Juzar Thingna,[1,2] Daniel Manzano,[1,3,4] and Jianshu Cao[1,2,*]

[1] *Massachusetts Institute of Technology, Chemistry Department. Cambridge, Massachusetts 02139, USA*
[2] *Singapore-MIT Alliance for Research and Technology (SMART) Centre, Singapore 138602*
[3] *Singapore University of Technology and Design, Engineering Product Development. 8 Somapah Road, Singapore 487372*
[4] *Universidad de Granada, Departamento de Electromagnetismo y Fsica de la Materia, and Instituto Carlos I de Física Teórica y Computacional, Granada 18071, Spain*

4-site model revisited

The results we show in the main text correspond to a system where diagonal and off-diagonal elements of the Hamiltonian H'_S are chosen to be the same. This requirement can be relaxed. Symmetries could exist even if these parameters are mismatched. In this section we explore a more general case with the help of the 4-site model and show that the signatures of molecular symmetries are robust. Thus, if the system possess a molecular symmetry in any form our probe based approach could help detect these symmetries. We begin by choosing a general 4-site Hamiltonian

$$H'_S = \sum_{i=1}^{4} \varepsilon_i |e_i\rangle\langle e_i| + \sum_{\langle i,j\rangle} h_{ij} |e_i\rangle\langle e_j|, \qquad (1)$$

where the parameters ε_i and $h_{ij} = h_{ji}$ are different for each site. In order to maintain the mirror symmetry of the system we choose $\varepsilon_2 = \varepsilon_4$, $h_{12} = h_{14}$, and $h_{23} = h_{34}$.

Figure 1 shows the behavior of the excitonic currents when the probe is placed either at site 1 (black solid lines) or at site 2 (green dashed-dotted lines) for symmetric (panel a), antisymmetric (panel b) or canonical (panel c) initial conditions. The conclusions remain the same as in the main text, i.e., the signatures of molecular symmetries is observed only in the case of a dark state initial condition. One main difference we observe when all parameters are not the same is the change in the dissipative Liouvillian spectrum (Fig. 1d). Contrary to the fully identical case (see Fig. 2 of main text) the eigenspectrum changes drastically. Although the splitting of the degenerate eigenvalue still persists and the unstable manifold still remains closest to the zero eigenvalue thus influencing the long relaxation times of the system.

Effect of probe position in para-Benzene

Similar to the 4-site model shown in the main text the effect of a local probe on benzene is shown in Fig. 2. The qualitative features remain the same as in the 4-site system with the anti-symmetric state showing the clear signatures of multiple steady states. The Liouvillian spectrum also shows exotic modes like oscillating coherences [1] that correspond to eigenvalues of the Liouvillian with zero real part but finite imaginary part. These could be the modes responsible for the observation of circular currents observed in benzene [2].

[1] Albert, V. V. & Jiang, L. Symmetries and conserved quantities in Lindblad master equations *Phys. Rev. A* **89**, 022118 (2014).

[2] Rai, D., Hod, O., & Nitzan, A. *C*ircular currents in molecular wires. *J. Phys. Chem. C* **114**, 20583 (2010).

* jianshu@mit.edu

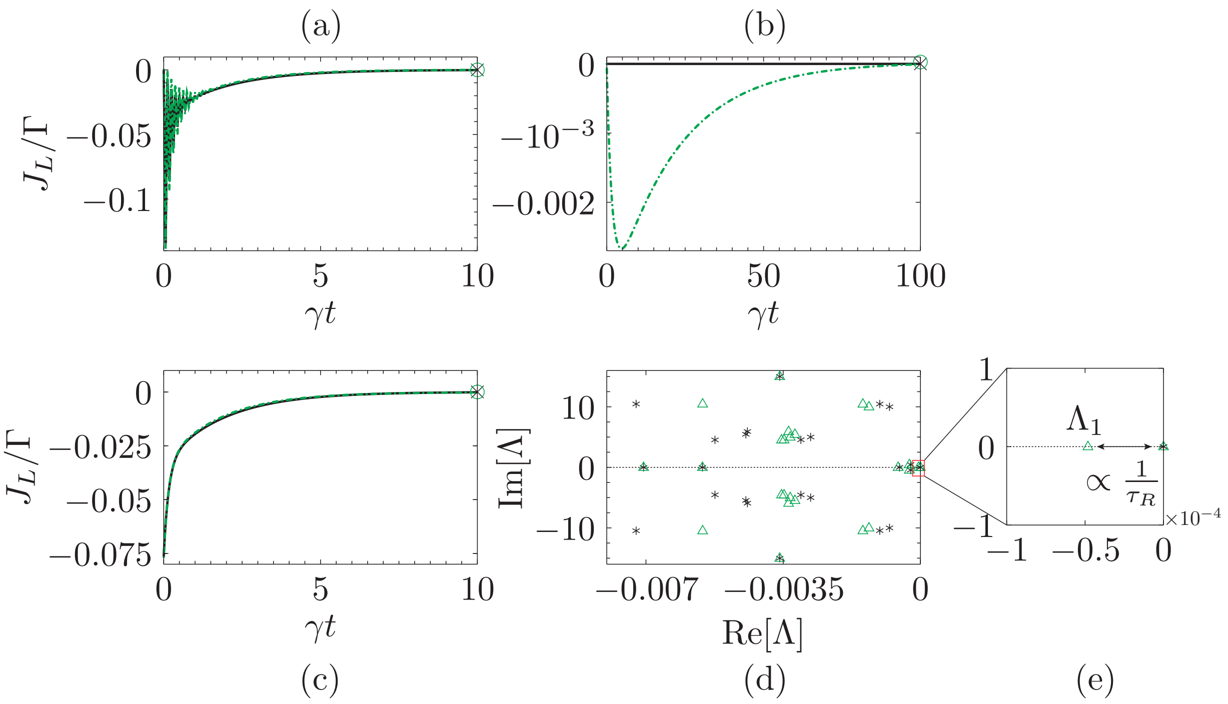

FIG. 1. **Effect of probe position on symmetry detection.** Time evolution of the excitonic current in the 4-site model with symmetric (panel a), antisymmetric (panel b), and canonical (panel c) initial conditions. (d) shows the eigenspectrum of the dissipative Liouvillian and (e) depicts the magnification around the zero eigenvalue. The dashed black line in panels (d) and (e) marks the $\mathrm{Im}[\Lambda] = 0$ axis. The probe is positioned at sites 1 (black solid line in panels a, b and c; black asterisk in panels d and e) and 2 (green dashed-dotted line in panels a, b, and c; green triangles in panels d and e) for all panels. The crosses and circles correspond to the nonequilibrium steady-state values of the excitonic currents. The system parameters are: $\varepsilon_1 = -64.6$meV, $\varepsilon_3 = -193.9$ meV, $\varepsilon_2 = \varepsilon_4 = -129.3$ meV, $h_{12} = h_{14} = -14.52$ meV, and $h_{23} = h_{34} = -1.59$meV. The lead and probe parameters are chosen as: $T_L = 330K$, $T_R = 270K$, $T = 300K$, $\Gamma = 196$GHz, $\gamma = 19.6$GHz, $\omega_0 = 78.55$THz, and $\omega_D = 1.96$ THz.

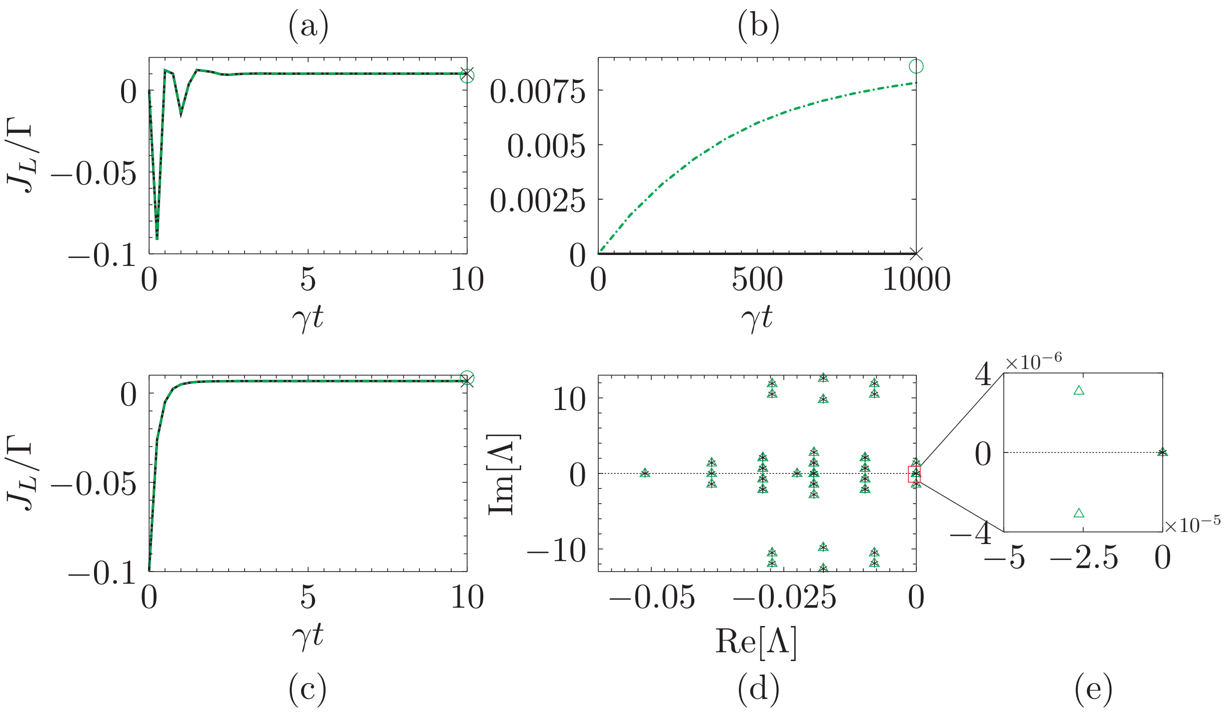

FIG. 2. **Effect of probe position on symmetry detection.** Time evolution of the excitonic current for the Benzene molecule with symmetric in sites 2 and 6 (panel a), antisymmetric in sites 2 and 6 (panel b), and canonical (panel c) initial conditions. The crosses and circles correspond to the nonequilibrium steady-state values of the excitonic currents. Panel (d) shows the eigenspectrum of the dissipative Liouvillan and (e) depicts the magnification around zero eigenvalue. The dashed black line in panels (d) and (e) marks the $\mathrm{Im}[\Lambda] = 0$ axis. The probe is positioned at sites 1 (black solid line panels a, b and c; black asterix in panels d and e) and 2 (green dashed-dotted line in panels a, b, and c; green triangles in panels d and e). The system parameters are: $\varepsilon = -11.2$eV and $h = -0.7$eV. The lead and probe parameters are chosen as: $T_L = 330K$, $T_R = 270K$, $T_P = 300K$, $\Gamma = 151.9$THz, $\gamma = 15.19$THz, $\omega_D = 151.92$THz and $\omega_0 = 78.55$THz.
